# Deep Learning for Multi-Tissue Segmentation and Fully Automatic Personalized Biomechanical Models from BACPAC Clinical Lumbar Spine MRI

**DOI:** 10.1093/pm/pnac142

**Published:** 2022-10-31

**Authors:** Madeline Hess, Brett Allaire, Kenneth T Gao, Radhika Tibrewala, Gaurav Inamdar, Upasana Bharadwaj, Cynthia Chin, Valentina Pedoia, Mary Bouxsein, Dennis Anderson, Sharmila Majumdar

**Affiliations:** Department of Radiology and Biomedical Imaging, Center for Intelligent Imaging, University of California, San Francisco, San Francisco, California; Center for Advanced Orthopedic Studies, Beth Israel Deaconess Medical Center, Harvard Medical School, Boston, Massachusetts, USA; Department of Radiology and Biomedical Imaging, Center for Intelligent Imaging, University of California, San Francisco, San Francisco, California; Department of Radiology and Biomedical Imaging, Center for Intelligent Imaging, University of California, San Francisco, San Francisco, California; Department of Radiology and Biomedical Imaging, Center for Intelligent Imaging, University of California, San Francisco, San Francisco, California; Department of Radiology and Biomedical Imaging, Center for Intelligent Imaging, University of California, San Francisco, San Francisco, California; Department of Radiology and Biomedical Imaging, Center for Intelligent Imaging, University of California, San Francisco, San Francisco, California; Department of Radiology and Biomedical Imaging, Center for Intelligent Imaging, University of California, San Francisco, San Francisco, California; Center for Advanced Orthopedic Studies, Beth Israel Deaconess Medical Center, Harvard Medical School, Boston, Massachusetts, USA; Center for Advanced Orthopedic Studies, Beth Israel Deaconess Medical Center, Harvard Medical School, Boston, Massachusetts, USA; Department of Radiology and Biomedical Imaging, Center for Intelligent Imaging, University of California, San Francisco, San Francisco, California

**Keywords:** Deep Learning, Magnetic Resonance Imaging, Musculoskeletal, Biomechanics, Quantitative Imaging, Lumbar Spine, Chronic Low Back Pain, BACPAC

## Abstract

**Study Design:**

In vivo retrospective study of fully automatic quantitative imaging feature extraction from clinically acquired lumbar spine magnetic resonance imaging (MRI).

**Objective:**

To demonstrate the feasibility of substituting automatic for human-demarcated segmentation of major anatomic structures in clinical lumbar spine MRI to generate quantitative image-based features and biomechanical models.

**Setting:**

Previous studies have demonstrated the viability of automatic segmentation applied to medical images; however, the feasibility of these networks to segment clinically acquired images has not yet been demonstrated, as they largely rely on specialized sequences or strict quality of imaging data to achieve good performance.

**Methods:**

Convolutional neural networks were trained to demarcate vertebral bodies, intervertebral disc, and paraspinous muscles from sagittal and axial T1-weighted MRIs. Intervertebral disc height, muscle cross-sectional area, and subject-specific musculoskeletal models of tissue loading in the lumbar spine were then computed from these segmentations and compared against those computed from human-demarcated masks.

**Results:**

Segmentation masks, as well as the morphological metrics and biomechanical models computed from those masks, were highly similar between human- and computer-generated methods. Segmentations were similar, with Dice similarity coefficients of 0.77 or greater across networks, and morphological metrics and biomechanical models were similar, with Pearson R correlation coefficients of 0.69 or greater when significant.

**Conclusions:**

This study demonstrates the feasibility of substituting computer-generated for human-generated segmentations of major anatomic structures in lumbar spine MRI to compute quantitative image-based morphological metrics and subject-specific musculoskeletal models of tissue loading quickly, efficiently, and at scale without interrupting routine clinical care.

## Introduction

Chronic lower back pain (cLBP) is a leading cause of disability in the United States [1] and is estimated to affect 540 million people worldwide [2]. Within the United States, cLBP is responsible for an estimated loss of 150 million workdays annually [3, 4] and is associated with an estimated annual cost of $100 to $200 billion [5, 6]. cLBP is one of the most common drivers of visits to a physician, but despite rapidly increasing treatment costs, patient outcomes have not improved substantially over time [7, 8]. cLBP is nonspecific in 62.2% of cases [9], making patient-specific treatment interventions difficult to develop. The etiology of cLBP is multifactorial and includes physical, psychological, environmental, and socioeconomic factors [10]. Challenges in isolating the causes of pain can lead to overuse of imaging, opioids, and surgical treatment options.

Magnetic resonance imaging (MRI) and other medical imaging techniques are often used to design and monitor treatment strategies for patients with cLBP. Imaging alone is a weak predictor of pain presence and pain drivers because of a lack of consistent associations between imaging studies and clinical symptoms [11, 12]. There is debate in the literature about the relationships between different image-based morphological metrics and spine-related health indicators [11–22], in part because of limited sample sizes and inconsistent measurement of imaging features across readers and institutions.

Segmentation of medical images is essential to unlocking scalable and reliable quantitative image-based markers of cLBP from spine morphology, but it can be prohibitively costly to perform. Manual segmentation is expensive and time intensive, requiring experienced readers between 15 minutes and several hours to annotate each exam, depending on the number of slices and structures of interest. Furthermore, despite the time and expertise investment required, annotations are subject to human error and bias across readers, which makes them difficult to generate and assess consistently in large-scale studies. Segmentation of the vertebral bodies, intervertebral discs, and paraspinous muscles in MRI can be valuable for diagnosing and characterizing spine degeneration and various pathologies related to cLBP, including stenosis, scoliosis, and osteoporosis [23]. Estimation of internal tissue loading demands is a promising potential indicator for evaluating subject-specific risks for back pain and injury prevention [24]. Tissue loading cannot be directly measured, but biomechanical musculoskeletal models based on medical images can be used to provide valid estimates of tissue loading [25]. Segmentation of images of the spine is an essential step to creating subject-specific musculoskeletal models, which improve on generic models by incorporating measurements like spine curvature and muscle morphology [26]. If segmentation can be performed in a fast, low-cost manner, personalized biomechanical models of patients could be automatically created as part of a clinical workflow. These models could then be used to evaluate measures of tissue loading as a component of back pain and to design patient-specific interventions.

Deep learning–based segmentation methods offer improvements in standardization and scalability when compared with human segmentation and can be leveraged to improve data collection in cLBP research. Combining computational methods with MRI presents opportunities for improved diagnosis, with potential for multifactorial analysis, quantitative analysis, and increased sample sizes.

We propose a framework for automatic segmentation of vertebral bodies, intervertebral discs, and paraspinous muscles in clinically acquired sagittal and axial T1-weighted MRIs of the lumbar spine and assess its efficacy as a substitute for manual segmentation in calculating intervertebral disc height, muscle cross-sectional area, and metrics of lumbar spine loading with subject-specific biomechanical models.

## Methods

This study was approved by the University of California, San Francisco (UCSF), Institutional Review Board. A retrospective clinical dataset of 206 MRI exams with both axial and sagittal T1-weighted acquisitions was aggregated as part of UCSF’s Back Pain Consortium. All exams were randomly selected from clinical scans at UCSF between 2008 and 2018. Of that set, 24/27/45 (vertebral body / disc / muscle) exams were annotated with the respective anatomic structure. Cases with bone fractures, extensive implants, primary tumors, and wide-spread metastatic disease to the spine were excluded. All volumes included were drawn from clinical exams obtained on GE scanners and followed the image acquisition parameters detailed in Table 1. Images were captured with subjects in head-first or feet-first supine position.

**Table 1.** Image acquisition parameters

MR Imaging Sequence	Acquisition Parameters	Associated Network
T1 sagittal	TR = 500–735 ms, TE = 8.184–21.1 ms, ETL = 2–6, Acquisition size = [0, 288, 224, 0]—[384, 0, 0, 224] Image size = 512 × 512, Resolution = 0.4688 × 0.4688 mm—0.5078 × 0.5078 mm, Slice thickness = 3.0–4.0 mm Space between slices = 3.0–5.0 mm	Vertebral body segmentation network Intervertebral disc segmentation network
T1 axial	TR = 503–983 ms, TE = 7.672–17.376 ms, ETL = 3–6, Acquisition size = [0, 256, 160, 0]—[320, 0, 0, 224] Image size = 256 × 256–512x512, Resolution = 0.3125 × 0.3125–0.7031 × 0.7031 mm, Slice thickness = 3.0–4.0 mm Space between slices = 3.0–5.0 mm	Muscle segmentation network

ETL = Echo train length; TE = Echo time; TR = Repetition time.

**Figure 1.**
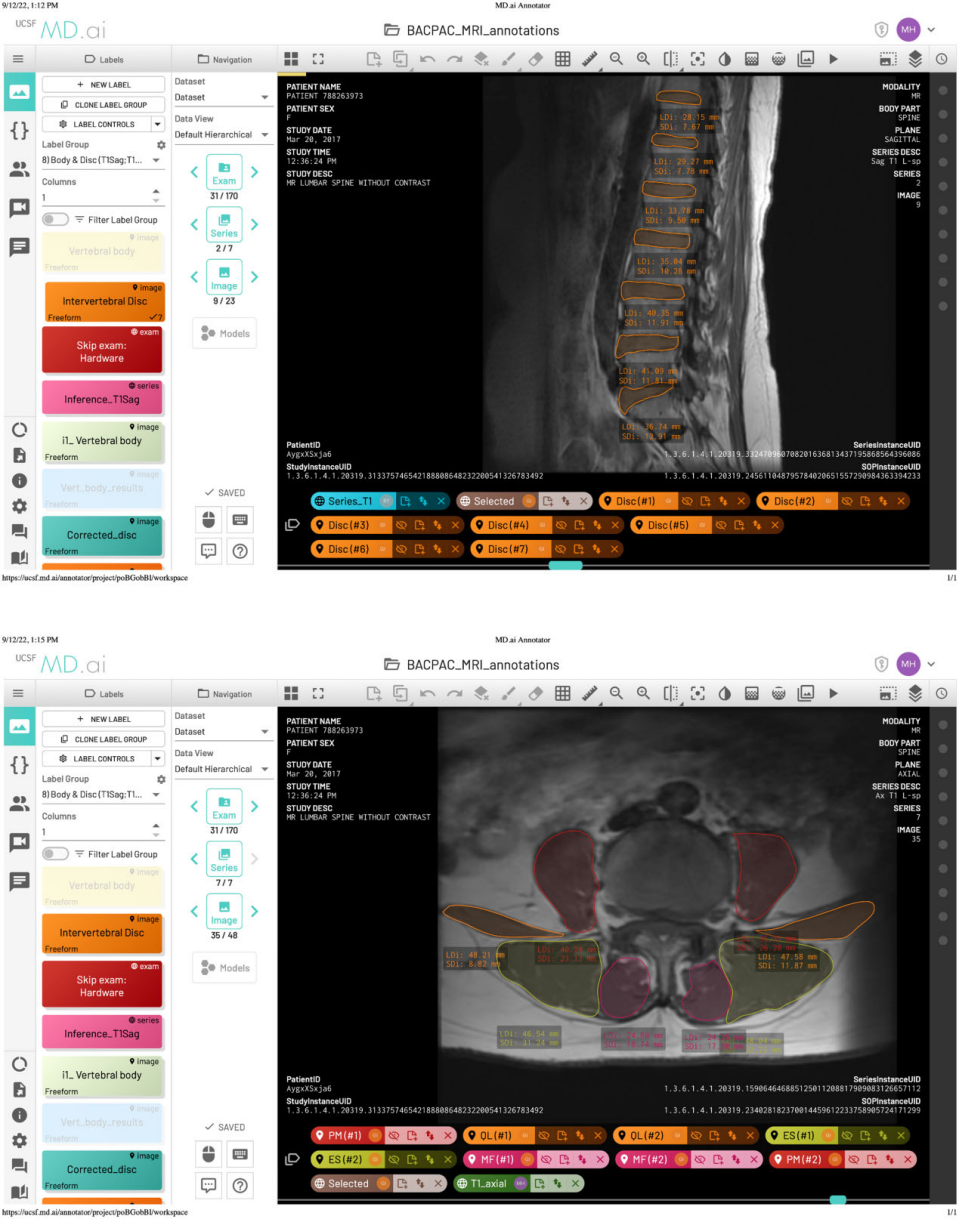
MD.ai annotation software. Each anatomic structure was annotated with a click-and-drag interface using the MD.ai annotation tool.

Manual vertebral body segmentations were performed on all slices in 24 exam volumes by one reader (GI). Intervertebral disc segmentations were similarly annotated on all slices in 27 exams by two readers (GI, MH). Paraspinal muscle segmentations were annotated on one to three slices at each lumbar disc level in 45 exams by one reader (GI). Both readers were trained by a board-certified radiologist (UB). All annotations were performed with MD.ai annotation software (MD.ai, New York, NY) (see Figure 1). When annotating the vertebral bodies and intervertebral discs, readers were instructed to segment all structures visible on every slice of a volume, excluding 1) any bodies and discs that were not completely pictured in the field of view and 2) structures inferior to the S1 vertebral body and L5–S1 intervertebral disc. All readers were trained to identify respective anatomy by a board-certified radiologist.

Separate 2D V-Nets [27] were trained to segment each anatomic structure on each sequence. The V-Net architecture and associated Dice-based loss function have previously been demonstrated to have improved performance and convergence time on medical image segmentation tasks over other popular network architectures. A 2D instead of 3D approach was selected to maximize use of available data, as the wide variation in number of annotated slices per exam would have required substantial slice-dimension coercion, which could impart bias through duplication or interpolation and loss of information through cropping. Because slice thickness in clinical images is greater than the in-plane resolution, segmentation of these sequences was a good candidate for a two-dimensional method. Two versions of each convolutional neural network were trained to segment the vertebral bodies, intervertebral discs, and paraspinous muscles, respectively, from T1-weighted MRI volumes. Two-version splitting was required to maximize both training performance and unbiased testing of biomechanical models; the standard split was constructed to demonstrate expected network performance under typical conditions, while the shared split was constructed to maximize samples in a hold-out test set to assess downstream performance without data leakage. The first version of each of the three models was computed with a standard random split of approximately 75% train, 15% validation, and 10% test to demonstrate optimal model performance on segmentation of each anatomic structure; we call this version the *standard split*. To accommodate limitations in data quantity, the networks were then retrained with new splits, which reserved a shared set of 10 patients across models for testing to assess the performance of this segmentation pipeline on automatic morphological metric and biomechanical model generation; we call this version the *shared split*. All networks were trained according to the hyperparameters listed in Table 2.

**Table 2.** Hyperparameters on all trained networks

	T1 Sagittal Disc Segmentation	T1 Sagittal Vertebral Body Segmentation	T1 Axial Muscle Segmentation
Standard split networks			
Network architecture	2D VNet	2D VNet	2D VNet
Input image size	512 × 512	512 × 512	256 × 256
Batch size	8 slices	8 slices	32 slices
Learning rate	1e^−4^	1e^−4^	1e^−4^
Dropout	0.2	0.05	0.2
Levels in network	4	3	3
Optimizer	Adam	Adam	Adam
Loss function	Weighted Dice sigmoid	Dice sigmoid	Weighted Dice sigmoid
Iterations until convergence	14,700	12,000	13,500
Shared split networks			
Network architecture	2D VNet	2D VNet	2D VNet
Input image size	512 × 512	512 × 512	256 × 256
Batch size	8 slices	8 slices	8 slices
Learning rate	1e^−4^	1e^−4^	1e^−4^
Dropout	0.2	0.05	0.2
Levels in network	4	3	4
Optimizer	Adam	Adam	Adam
Loss function	Weighted Dice sigmoid	Dice sigmoid	Weighted Dice sigmoid
Iterations until convergence	17,500	11,900	12,000

Morphological metrics and biomechanical models were evaluated only for the networks in the shared split. Intervertebral disc, vertebral body, and paraspinous muscle segmentations were inferred for each patient in the shared hold-out test set of 10 exams, through the use of the respective V-Net. Postprocessing was applied to all segmentations to smooth edges, fill holes, and isolate the largest connected components. Intervertebral disc height (IVDH), muscle cross-sectional area (CSA), and 2D and 3D centroid positions for each anatomic structure in patient-based space were then extracted from the segmentations. These quantitative features were then leveraged as inputs to construct subject-specific biomechanical models of the lumbar spine to extract measures of compressive loading on the vertebral bodies.

**Figure 2.**
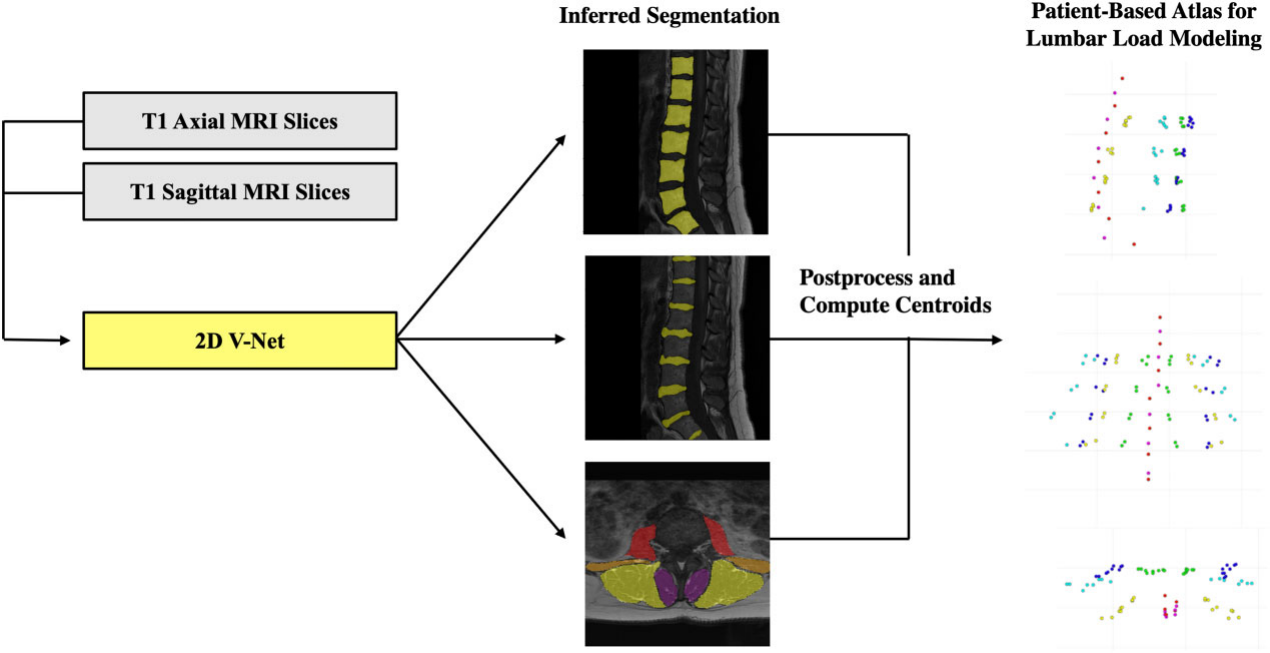
Visualization of centroid construction. T1 axial and T1 sagittal MRI slices were input into each respective V-Net to generate inferred segmentation masks of the vertebral bodies, intervertebral discs, and paraspinous muscles. After postprocessing, centers of mass were computed on each segmentation mask to calculate the position of volume-wise centroids for each vertebral body and intervertebral disc and slice-wise centroids for each paraspinous muscle. These centroids were then converted to patient-based space, yielding a 3D atlas of the lumbar spine for further biomechanical modeling.

To calculate IVDH, a 3D centroid was computed on each segmented disc to identify its most central slice; a minimum bounding rectangle was then constructed around the segmentation on the center slice to extract the shortest side length as a final height. Muscle CSA was constructed by calculating the sum of foreground pixels for each muscle and then multiplying by pixel spacing to yield area in square centimeters. Finally, a center of mass was computed in 3D to identify volume-wise centroids on each vertebral body and intervertebral disc and in 2D to identify slice-wise centroids on each muscle (see Figure 2). Each centroid point was then mapped to the patient-based coordinate system with an affine matrix transformation by using the source exam’s metadata, yielding results measured in distance from the scanner reference point.

Subject-specific musculoskeletal models of the trunk were created with vertebral body centroids, muscle centroids, and muscle CSA in OpenSim (SimTK) [28]. Starting with an appropriate sex-specific base model, models were scaled to the subject’s height and weight. Vertebral centroid locations and muscle morphology parameters were then incorporated into the model via custom Matlab 2019b (The MathWorks Inc., Natick, MA) [29] scripts to build the final model with subject-specific spine curvature and trunk muscle properties [26]. Lumbar spine compressive loading was evaluated with these models for a forward flexion activity (60 degrees of trunk flexion with 5-kg weights in each hand) at L1–L5 for each patient in the shared test set.

A volumetric Sørensen-Dice similarity coefficient [30] was calculated to assess the overall performance of each of the three networks. Each of the morphological metrics and biomechanical model outcomes was calculated on both the manual and inferred segmentations for each patient, creating results that could be directly compared to assess the effectiveness of substituting manual for automatic annotation. Bland-Altman plots and Pearson R correlation coefficients were computed to assess the relationships among IVDH, CSA, and loading metrics generated from manually vs automatically segmented data.

Visual inspection for quality control after model training revealed several errors. Errors on the vertebral body annotations included segmentation of the S2 vertebral body or lower, exclusion of lateral slices in which bone was visible, and segmentation of both the vertebral body and bony pedicles in some of exams. One exam was annotated with paraspinous muscles on all slices. Major annotation errors were identified on one exam after training; the exam had been incompletely annotated and included fewer than half of the intervertebral discs, and it was consequently excluded from the hold-out test set on the shared split model.

## Results

**Figure 3.**
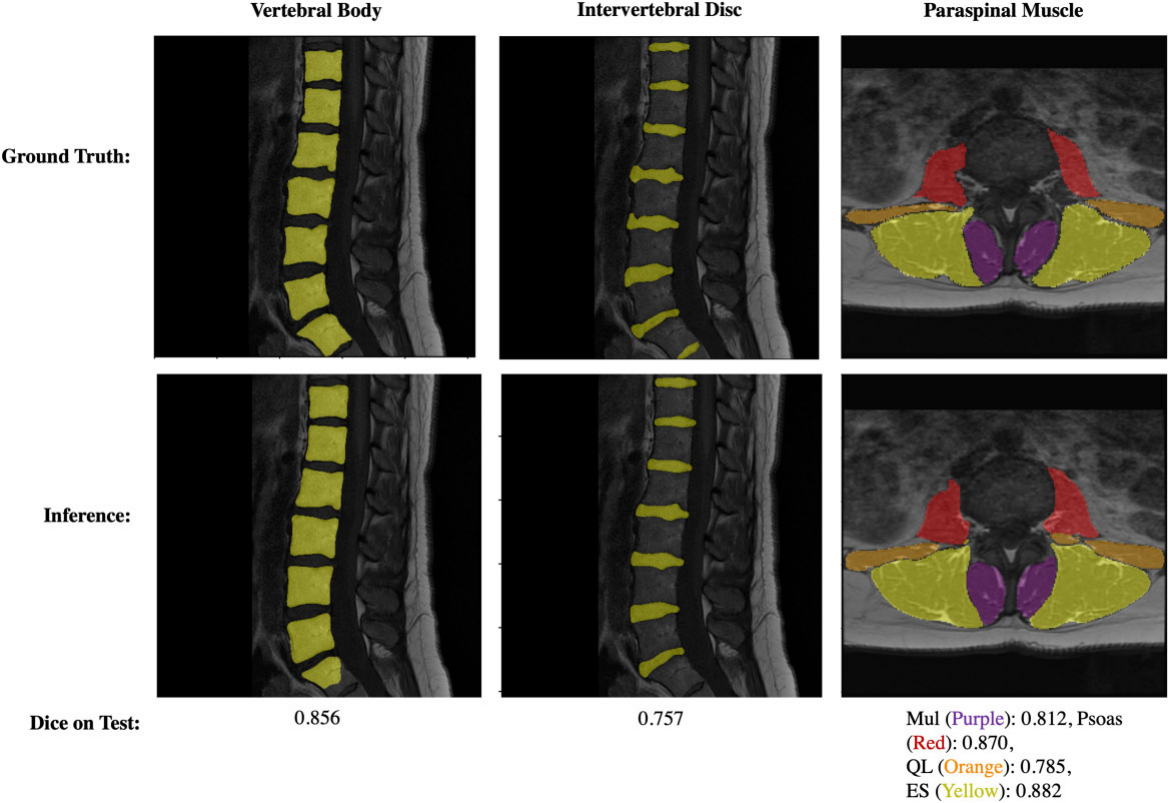
Visualization of segmentation results from each network. The first, second, and third columns show examples of vertebral body, intervertebral disc, and paraspinal muscle segmentation results, respectively, along with a 3D Dice coefficient of each network’s performance.

**Figure 4.**
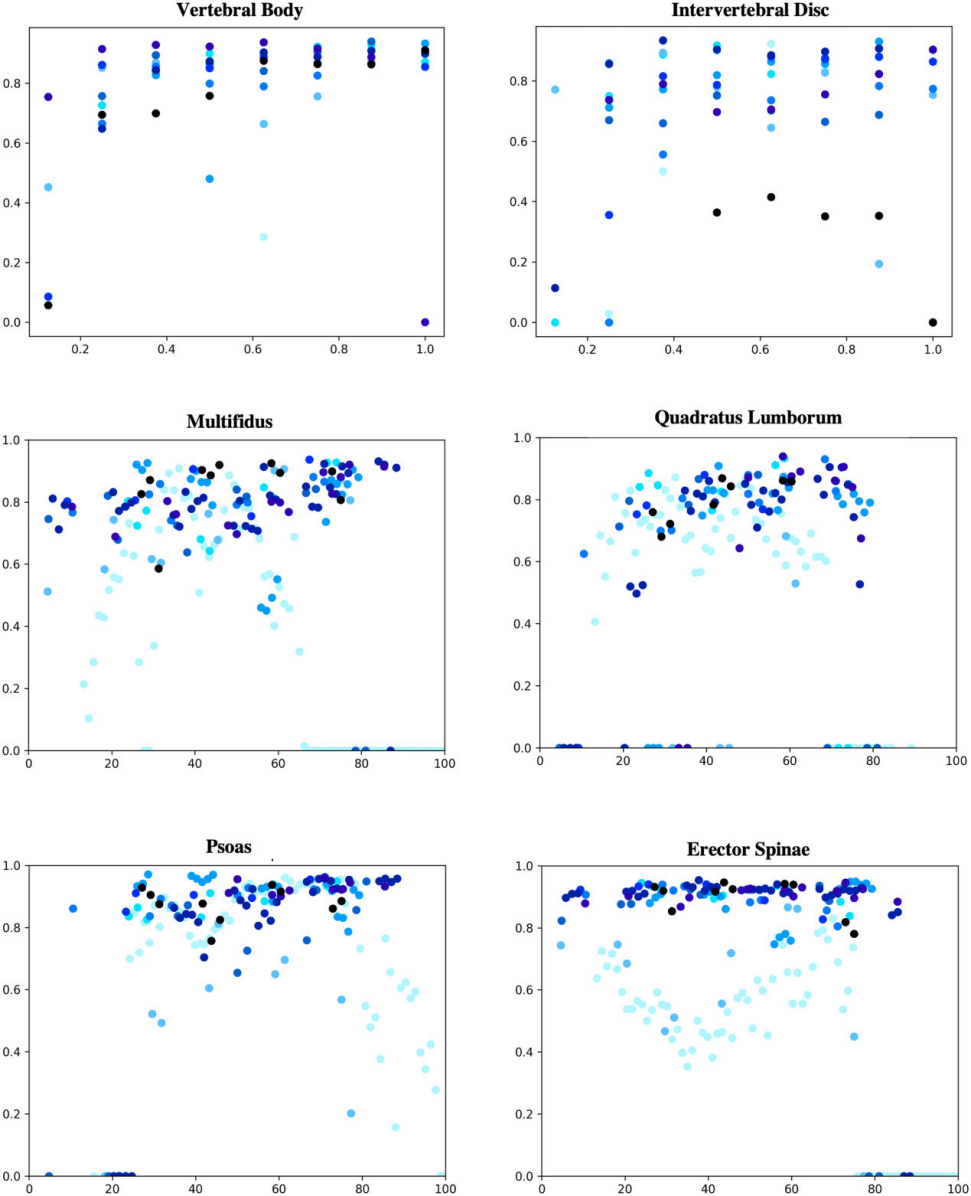
Stratified network performance (shared split). Vertebral body network performance at each vertebral body level **(top left)**. Intervertebral disc network performance at each disc level **(top right)**. Paraspinal muscle network performance at each slice level **(middle, bottom)**. Note that the number of slices in each volume was normalized to a shared size, and each slice index was adjusted accordingly, to enable a visual comparison across patients. Performance was measured with spatial Dice. Different shades of blue indicate different exams.

Volumetric Dice coefficients for each segmentation method are summarized in Tables 3, 4, and 5. The three segmentation networks with standard splits performed with Dice coefficients of 0.87 for the intervertebral disc network, 0.88 for the vertebral body network, and 0.81/0.95/0.84/0.92 (*mul*/*psoas*/*QL*/*ES*) for the muscle network on each respective test set. After re-splitting and retraining to construct a set of the shared split networks, Dice coefficients decreased to 0.86 on disc, 0.76 on vertebral body, and 0.81/0.87/0.79/0.88 (*mul*/*psoas*/*QL*/*ES*) on muscle on the shared test set. Manual segmentations of the same exam demarcated by different annotators showed Dice coefficient similarities of 0.83 on disc, 0.93 on vertebral body, and 0.78 or greater on muscle. Manual segmentations of the same exam from one reader after a washout period of at least 3 weeks showed similar Dice coefficients of 0.88 on disc, 0.95 on vertebral body, and 0.87 or greater on muscle. Example images of manual and automatic segmentations are shown in Figure 3. The vertebral body network performed with level-wise volumetric Dice coefficients greater than or equal to its overall performance of 0.76 on all levels. Level-wise performance on the intervertebral disc network dropped below its overall performance of 0.86 on all disc levels. Results on the S2 vertebral body and S1S2 intervertebral disc are not reported, as annotators were instructed to exclude the S2 vertebral body and S1S2 intervertebral disc. Stratified volumetric Dice coefficients could not be computed because of the absence of objective markers with which to partition axial slices into level-based groups, given that the annotators were not required to segment each slice. Instead, two-dimensional slice-wise Dice coefficients were used to measure stratified network performance for each muscle group in the paraspinous muscle network (Figure 4).

**Table 3.** Overall segmentation network performance on hold-out test set

Network	Intra-Reader (n)	Inter-Reader (n)	Standard Split (n)	Shared Split (n)
T1 sagittal intervertebral disc	0.88 ± 0.056 (3)	0.83 ± 0.039 (3)	0.87 ± 0.13 (2)	0.81 ± 0.047 (9)
T1 sagittal vertebral body	0.95 ± 0.013 (3)	0.93 ± 0.025 (3)	0.82 (1)	0.86 ± 0.033 (10)
T1 axial paraspinous muscle				
Multifidus	0.88 ± 0.065 (3)	0.87 ± 0.049 (3)	0.81 ± 0.11 (2)	0.78 ± 0.082 (10)
Psoas	0.94 ± 0.026 (3)	0.93 ± 0.028 (3)	0.95 ± 0.053 (2)	0.86 ± 0.078 (10)
Quadratus lumborum	0.79 ± 0.19 (3)	0.76 ± 0.21 (3)	0.84 ± 0.29 (2)	0.77 ± 0.078 (10)
Erector spinae	0.92 ± 0.028 (3)	0.92 ± 0.029 (3)	0.92 ± 0.24 (2)	0.84 ± 0.092 (10)

**Figure 5.**
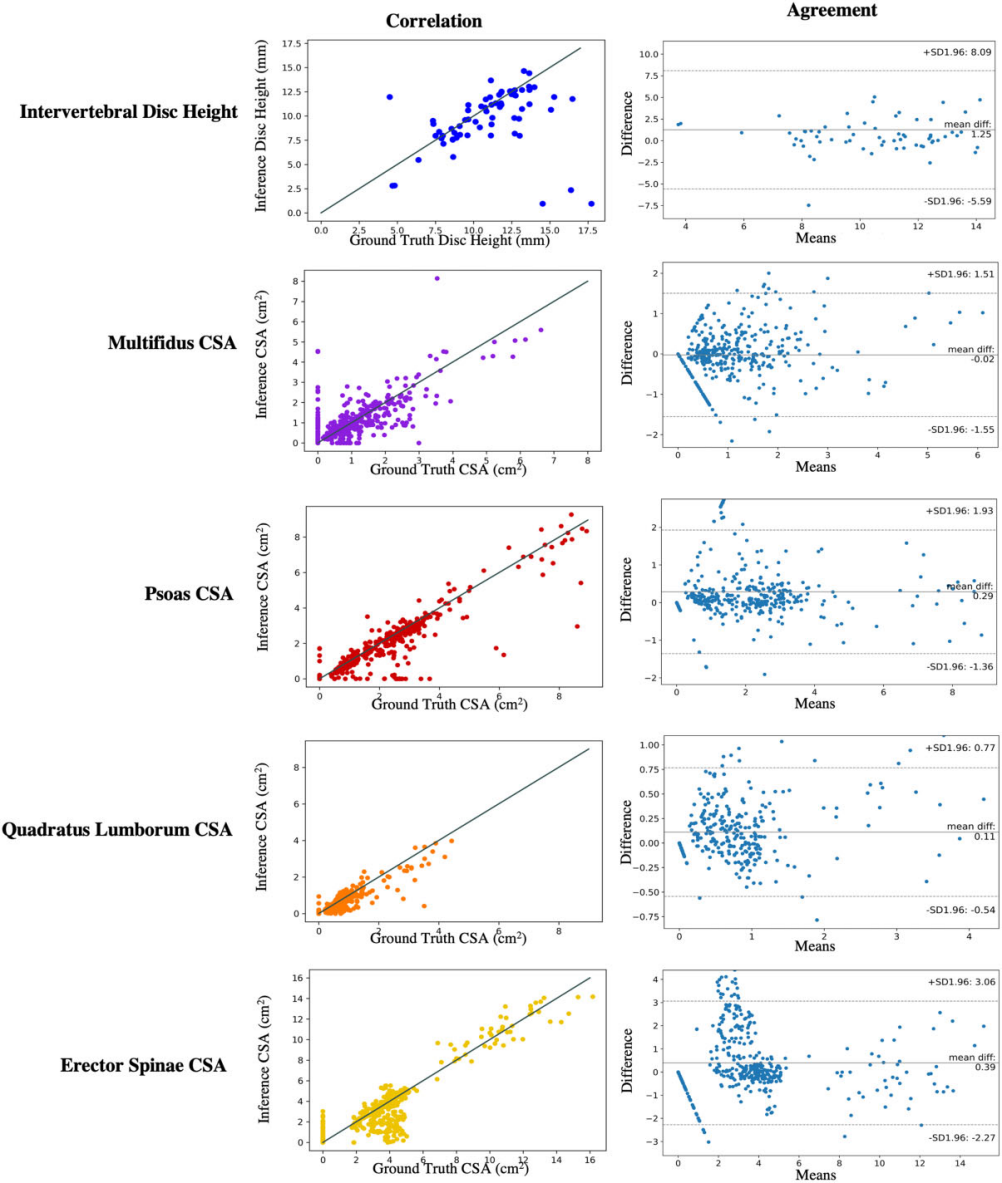
Correlation **(left)** and agreement **(right)** between manually and automatically generated segmentations for each biomarker. Correlation between disc height from manual vs inferred disc segmentations is displayed on a scatter plot, where the line x=y is indicated in grey. Agreement is displayed with Bland-Altman plots for disc height on each disc.

**Figure 6.**
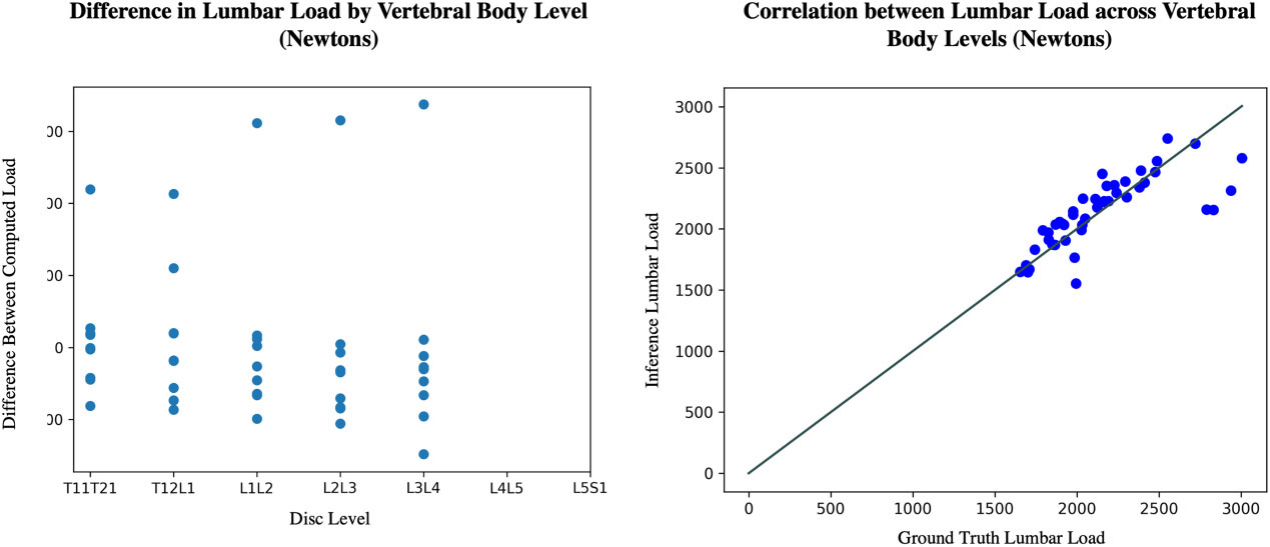
Stratified loading performance. Difference **(left)** and correlation **(right)** between loading metrics generated from human- vs computer-generated segmentation masks, stratified by vertebral body level.

Pearson R correlation coefficients and mean absolute error were used to measure the relationship between morphological metrics calculated from manually vs automatically generated segmentations (Tables 6, 7, and 8). IVDH calculated from network-generated segmentations vs manually generated segmentations showed a correlation coefficient of 0.26 and a mean absolute error of 1.98 mm between the two methods. Muscle CSA from automatic segmentation was correlated with that of manual segmentations with coefficients of 0.74/0.850.70/0.37 and mean absolute errors of 1.21/1.36/0.82/3.55 cm^2^ (*mul*/*psoas*/*QL*/*ES*). Centroid locations differed, with mean absolute errors of 3.03/3.64/7.23/3.58 mm in Euclidean distance between ground truth and inference. Vertebral body compressive loading computed between inferred and manually generated input data was correlated with coefficients 0.93/0.90/0.78/0.68/0.52 (L1/L2/L3/L4/L5). Bland-Altman and scatter plots for manually vs automatically generated intervertebral disc height and muscle CSA show correlation and agreement between the two methods (see Figures 5 and 6).

**Table 4.** Level-stratified vertebral body network performance (shared split, n = 10)

Level	Shared Split	Intra-Reader	Inter-Reader
	± 95% CI	± 95% CI	± 95% CI
T11	0.0243	0.947	0. 956
T12	0.888 ± 0.0218	0.968 ± 0.0113	0.957 ± 0.0252
L1	0.900 ± 0.0529	0.972 ± 0.0129	0.955 ± 0.0340
L2	0.888 ± 0.0642	0.971 ± 0.00449	0.939 ± 0.0341
L3	0.902 ± 0.0247	0.973 ± 0.00616	0.943 ± 0.00634
L4	0.863 ± 0.0753	0.972 ± 0.00542	0.951 ± 0.00957
L5	0.865 ± 0.0501	0.959 ± 0.0334	0.940 ± 0.0710
S1	0.684 ± 0.203	0.957 ± 0.0087	0.927 ± 0.162

**Table 5.** Level-stratified intervertebral disc network performance (shared split, n = 9)

Level	Shared Split	Intra-Reader	Inter-Reader
	± **95% CI**	± 95% CI	± 95% CI
T11T12	0.54 ± 0.311	0.867	0.791
T12L1	0.844 ± 0.0567	0.886 ± 0.0165	0.864 ± 0.0471
L1L2	0.731 ± 0.213	0.848 ± 0.0850	0.799 ± 0.127
L2L3	0.813 ± 0.0586	0.868 ± 0.173	0.654 ± 0.804
L3L4	0.730 ± 0.220	0.908 ± 0.018	0.582 ± 1.25
L4L5	0.711 ± 0.216	0.871 ± 0.142	0.831 ± 0.116
L5S1	0.650 ± 0.200	0.899 ± 0.0470	0.554 ± 1.19

**Table 6.** Overall performance of manually vs automatically generated morphometrics

Biomarker	Pearson R (*P* Value)	Mean Absolute Error
Intervertebral disc height, mm	0.846 (2.48 × 10^–18^)	1.45 ± 0.380
Muscle CSA—multifidus, cm^2^	0.720 (3.50 × 10^–63^)	0.489 ± 0.122
Muscle CSA—psoas, cm^2^	0.892 (2.36 × 10^–135^)	0.457 ± 0.152
Muscle CSA—quadratus lumborum	0.905 (1.34 × 10^–145^)	0.197 ± 0.0584
Muscle CSA—erector spinae	0.897 (4.08 × 10^–139^)	0.964 ± 0.208
Lumbar loading, Newtons	0.767 (8.29 × 10^–10^)	143 ± 50.3

**Table 7.** Stratified intervertebral disc height calculation performance (n = 9)

Level	Pearson R (*P* Value)	Mean Absolute Error (mm)
All levels	0.846 (2.48 × 10^–18^)	1.45 ± 0.38
T11T12/T12L1	0.951 (8.02 × 10^–05^)	1.05 ± 1.03
T12L1/L1L2	0.847 (0.00396)	1.23 ± 0.85
L1L2/L2L3	0.155 (0.69)	2.17 ± 1.81
L2L3/L3L4	0.696 (0.0372)	0.90 ± 0.52
L3L4/L4L5	0.795 (0.0105)	1.24 ± 1.02
L4L5/L5L6	0.562 (0.115)	2.24 ± 1.26
L5S1/L6S1	0.945 (0.000124)	1.32 ± 1.18

## Discussion

Previous studies have demonstrated the viability of automatic segmentation applied to medical images but have left notable gaps in the problem space in designing methods that integrate seamlessly into clinical workflow and create pathways to apply the technology at scale to biomechanical research. Demonstration of the feasibility of these networks to segment clinically acquired images is limited, instead requiring a non-routine protocol or strict quality controls [23, 31–33]. Published networks do not apply this technology across planes and views to segment multiple anatomic structures or construct a fully subject-specific 3D atlas of the lumbar spine [34]. Additionally, published networks are specialized to downstream anomaly detection, not biomechanical modeling [35]. As a result, clinical translation of these methods is unfeasible on a large scale, as adding specialized imaging sequences to existing clinical protocols is costly and time intensive to institutions and patients. The generalizability of these methods to imaging studies routinely acquired in clinical settings is not yet proven.

We demonstrate the feasibility of substituting manual with automatic segmentation of the vertebral bodies, intervertebral discs, and paraspinous muscles by using networks trained on a small amount of clinical data. Overall segmentation network performance indicates that manual and automatic segmentation methods perform similarly, and morphological metric calculation can largely be outsourced to neural networks. Although stratified performance results indicate value in human oversight of network performance and morphological metric generation, there are clear efficiency gains within an acceptable margin of error to be found by implementing fully automatic assistance when delineating vertebral bodies, intervertebral discs, and paraspinous muscle in T1-weighted MRI. The proposed segmentation pipeline and the associated quantitative feature generation methods have applications in both a clinical and a research context, as they will enable researchers to analyze larger datasets of potential biomarkers of cLBP and quickly provide those same features to clinicians to improve disease characterization and treatment in real time.

Performance for both the vertebral body and intervertebral disc segmentation networks is strongest on the central-most lumbar levels, which was consistent variation in training data and anatomic boundaries. Because of natural variation in patient anatomy, exams in model training exhibited a range of numbers of vertebral bodies and intervertebral discs in the lumbar spine. Annotators were instructed to exclude vertebral bodies that were not completely pictured in the field of view, as well as to exclude the S2 vertebral body or the S1S2 intervertebral disc, but features of vertebral body and intervertebral disc boundaries are similar across the lumbar spine. This led to a pattern in which trained networks inconsistently segmented the most superior and most inferior vertebral bodies and intervertebral discs in the field of view, as networks were arbitrarily penalized for correctly identifying these bodies in training. This phenomenon is reflected in the network’s relatively poorer performance when segmenting the S2 vertebral body and T11T12 and S1S2 intervertebral discs (Table 3). Segmentation of the multifidus, psoas, and erector spinae performance variance has no demonstrated correlation with slice level in most exams. Segmentation performance on the quadratus lumborum tends to drop on the most inferior and superior slices, consistent with anatomic expectation above L1 and below L5 (Figure 4).

Error trends in automatic calculation of intervertebral disc height reflected trends in model error on the disc segmentation network, with statistical significance occurring only at the T12L1/L1L2 level. Results of all levels except L2 and L3 were inconclusive in the lumbar loading comparison. The decline in correlation from L1 to L5 could suggest greater sensitivity of lower lumbar loads to the subject-specific model inputs, as models generate less reliable results on slices at the boundaries of anatomy. Automatic segmentation as an input might have tended to underestimate the height of each disc and load per vertebral body, but conclusions cannot be drawn given the small sample size.

We present a highly scalable, fully automatic framework to generate quantitative measures of spine morphology and subject-specific biomechanical models from lumbar spine MRI. We demonstrate that results generated by this pipeline are highly correlated and agree with those generated by human readers, without human-in-the-loop correction. This work indicates that computer-generated segmentations could successfully substitute for human-demarcated masks, which are time intensive and costly to obtain, in the quantification of metrics of lumbar spine morphology and biomechanical models to quantify tissue loading. The networks used to create the predicted segmentations were trained on clinical exams with standard diagnostic sequences, which suggests strong generalizability with no extra costs associated with exam acquisition. A human-in-the-loop system to catch failures but improve time to acquire each segmentation could be of value to account for variation in performance with scan quality. A fully automatic, quantitative method for generating image-based features of spine morphology and validated estimates of tissue loading from clinically acquired MR exams combined with biomechanical modeling, like this one, would provide a scalable approach with which to evaluate drivers of cLBP across patients, institutions, and imaging archives without interrupting routine care.

## Supplementary Data

Supplementary Data may be found online at http://painmedicine.oxfordjournals.org.

**Table 8.** Lumbar load performance (n = 9)

Level	Pearson R (*P* Value)	Mean Absolute Error (Newtons)
All levels	0.767 (8.29 × 10^–10^)	143.26 ± 50.25
L1	0.699 (0.0361)	101.0 ± 104.45
L2	0.869 (0.00234)	136.66 ± 98.7
L3	0.795 (0.0105)	142.86 ± 146.32
L4	0.688 (0.0405)	163.64 ± 145.5
L5	0.547 (0.127)	172.14 ± 160.08

## Funding

Funding was received from the U.S. National Institutes of Health (UH2AR076724, R01-AR073019).

*Conflicts of interest:* All authors have no conflicts of interest to disclose.

## Supplement sponsorship

This article appears as part of the supplement entitled “Back Pain Consortium (BACPAC) Research Program” supported by the National Institutes of Health through the NIH HEAL Initiative under award number AR076730-01.

## Disclaimer

The content is solely the responsibility of the authors and does not necessarily represent the official views of the National Institutes of Health or its NIH HEAL Initiative.

## Supplementary Material

pnac142_Supplementary_Data

## References

[1] Centers for Disease Control and Prevention (CDC). Prevalence of disabilities and associated health conditions among adults—United States, 1999. MMWR Morb Mortal Wkly Rep 2001;50:120–5. 11393491

[2] Hartvigsen J , Hancock MJ, Kongsted A, et al What low back pain is and why we need to pay attention. Lancet 2018;391(10137):2356–67. 29573870 10.1016/S0140-6736(18)30480-X

[3] Guo HR , Tanaka S, Cameron LL, et al Back pain among workers in the United States: National estimates and workers at high risk. Am J Ind Med 1995;28(5):591–602. 8561169 10.1002/ajim.4700280504

[4] Guo HR , Tanaka S, Halperin WE, Cameron LL. Back pain prevalence in US industry and estimates of lost workdays. Am J Public Health 1999;89(7):1029–35. 10394311 10.2105/ajph.89.7.1029 PMC1508850

[5] Frymoyer JW , Cats-Baril WL. An overview of the incidences and costs of low back pain. Orthop Clin North Am 1991;22(2):263–71. 1826550

[6] Katz JN. Lumbar disc disorders and low-back pain: Socioeconomic factors and consequences. JBJS 2006;88:21–4. 10.2106/JBJS.E.01273 16595438

[7] Carey TS , Garrett J, Jackman A, et al The outcomes and costs of care for acute low back pain among patients seen by primary care practitioners, chiropractors, and orthopedic surgeons. N Engl J Med 1995;333(14):913–7. 7666878 10.1056/NEJM199510053331406

[8] Friedly J , Chan L, Deyo R. Increases in lumbosacral injections in the Medicare population: 1994 to 2001. Spine 2007;32(16):1754–60. 17632396 10.1097/BRS.0b013e3180b9f96e

[9] Iizuka Y , Iizuka H, Mieda T, et al Prevalence of chronic nonspecific low back pain and its associated factors among middle-aged and elderly people: An analysis based on data from a musculoskeletal examination in Japan. Asian Spine J 2017;11(6):989–97. 29279756 10.4184/asj.2017.11.6.989 PMC5738322

[10] Rubin DI. Epidemiology and risk factors for spine pain. Neurol Clin 2007;25(2):353–71. 17445733 10.1016/j.ncl.2007.01.004

[11] Steffens D , Hancock MJ, Maher CG, et al Does magnetic resonance imaging predict future low back pain? A systematic review. Eur J Pain 2014;18(6):755–65. 24276945 10.1002/j.1532-2149.2013.00427.x

[12] Steffens D , Hancock MJ, Pereira LSM, et al Do MRI findings identify patients with low back pain or sciatica who respond better to particular interventions? A systematic review. Eur Spine J 2016;25(4):1170–87. 26329648 10.1007/s00586-015-4195-4

[13] Lidar Z , et al Intervertebral disc height changes after weight reduction in morbidly obese patients and its effect on quality of life and radicular and low back pain. Spine 2012;37:1947–52. 22648024 10.1097/BRS.0b013e31825fab16

[14] Teichtahl AJ , Urquhart DM, Wang Y, et al Modic changes in the lumbar spine and their association with body composition, fat distribution and intervertebral disc height—a 3.0 T-MRI study. BMC Musculoskelet Disord 2016;17:92. 26891686 10.1186/s12891-016-0934-x PMC4759726

[15] Roberts N , Gratin C, Whitehouse GH. MRI analysis of lumbar intervertebral disc height in young and older populations. J Magn Reson Imaging 1997;7(5):880–6. 9307915 10.1002/jmri.1880070517

[16] D’hooge R , et al Increased intramuscular fatty infiltration without differences in lumbar muscle cross-sectional area during remission of unilateral recurrent low back pain. Man Ther 2012;17:584–8. 22784801 10.1016/j.math.2012.06.007

[17] Niemeläinen R , Briand M-M, Battié MC. Substantial asymmetry in paraspinal muscle cross-sectional area in healthy adults questions its value as a marker of low back pain and pathology. Spine 2011;36:2152–7. 21343855 10.1097/BRS.0b013e318204b05a

[18] Kim S-Y. Changes in cross-sectional area of lumbar muscle in patients with chronic back pain. J Korean Phys Ther 2010;22:39–47.

[19] Barker KL , Shamley DR, Jackson D. Changes in the cross-sectional area of multifidus and psoas in patients with unilateral back pain: The relationship to pain and disability. Spine 2004;29(22):E515–9. 15543053 10.1097/01.brs.0000144405.11661.eb

[20] Rezazadeh F , Taheri N, Okhravi SM, Hosseini SM. The relationship between cross-sectional area of multifidus muscle and disability index in patients with chronic non-specific low back pain. Musculoskelet Sci Pract 2019;42:1–5. 30981101 10.1016/j.msksp.2019.03.005

[21] Gwak G-T , Hwang U-J, Jung S-H, et al Comparison of MRI cross-sectional area and functions of core muscles among asymptomatic individuals with and without lumbar intervertebral disc degeneration. BMC Musculoskelet Disord 2019;20(1):576. 31787092 10.1186/s12891-019-2960-y PMC6886205

[22] Lee HJ , Lim WH, Park J-W, et al The relationship between cross sectional area and strength of back muscles in patients with chronic low back pain. Ann Rehabil Med 2012;36(2):173–81. 22639740 10.5535/arm.2012.36.2.173 PMC3358672

[23] Suzani A , Rasoulian A, Seitel A, et alDeep learning for automatic localization, identification, and segmentation of vertebral bodies in volumetric MR images. In: Webster III R, Yaniv Z, eds. Medical Imaging 2015: Image-Guided Procedures, Robotic Interventions, and Modeling. Vol. 9415 941514. Orlando, FL: International Society for Optics and Photonics; 2015.

[24] Dreischarf M , Shirazi-Adl A, Arjmand N, Rohlmann A, Schmidt H. Estimation of loads on human lumbar spine: A review of in vivo and computational model studies. J Biomech 2016;49(6):833–45. 26873281 10.1016/j.jbiomech.2015.12.038

[25] Mokhtarzadeh H , Anderson DE, Allaire BT, Bouxsein ML. Patterns of load-to-strength ratios along the spine in a population-based cohort to evaluate the contribution of spinal loading to vertebral fractures. J. Bone Miner Res 2021;36(4):704–11. 33253414 10.1002/jbmr.4222 PMC8383210

[26] Bruno AG , Mokhtarzadeh H, Allaire BT, et al Incorporation of CT-based measurements of trunk anatomy into subject-specific musculoskeletal models of the spine influences vertebral loading predictions. J Orthop Res 2017;35(10):2164–73. 28092118 10.1002/jor.23524 PMC5511782

[27] Milletari F , Navab N, Ahmadi S-A. V-Net: Fully convolutional neural networks for volumetric medical image segmentation. *arXiv:1606.04797 cs.CV*, 2016.

[28] Delp SL , Anderson FC, Arnold AS, et al OpenSim: open-source software to create and analyze dynamic simulations of movement. IEEE Trans Biomed Eng 2007;54(11):1940–50. 18018689 10.1109/TBME.2007.901024

[29] MATLAB. Version *9.7.0.1190202 (R2019b)*. Natick, MA: The MathWorks Inc.; 2018.

[30] Sørensen T. A Method of Establishing Groups of Equal Amplitude in Plant Sociology Based on Similarity of Species and Its Application to Analyses of the Vegetation on Danish Commons. København, DK: The Royal Danish Academy of Sciences and Letters; 1948.

[31] Zhou J , Damasceno PF, Chachad R, et al Automatic vertebral body segmentation based on deep learning of Dixon images for bone marrow fat fraction quantification. Front Endocrinol 2020;11:612. 10.3389/fendo.2020.00612 PMC7492292 32982989

[32] Xia W , Fortin M, Ahn J, et alAutomatic paraspinal muscle segmentation in patients with lumbar pathology using deep convolutional neural network. In: Shen D, et al, eds. Medical Image Computing and Computer Assisted Intervention – MICCAI 2019. Shenzhen, CN: Springer International Publishing, 2019:318–25 (doi: 10.1007/978-3-030-32245-8_36).

[33] Iriondo C , Pedoia V, Majumdar S. Lumbar intervertebral disc characterization through quantitative MRI analysis: An automatic voxel‐based relaxometry approach. Magn Reson Med 2020;84(3):1376–90. 32060963 10.1002/mrm.28210 PMC7318328

[34] Guinebert S , Petit E, Bousson V, et al Automatic semantic segmentation and detection of vertebras and intervertebral discs by neural networks. Comput Methods Programs Biomed Update 2022;2:100055.

[35] LewandrowskI K-U , Muraleedharan N, Eddy SA, et al Feasibility of deep learning algorithms for reporting in routine spine magnetic resonance imaging. Int J Spine Surg 2020;14(s3):S86–S97. 33298549 10.14444/7131 PMC7735442

