# Supplementary material for: Deep Learning for Multi-Tissue Segmentation and Fully Automatic Personalized Biomechanical Models from BACPAC Clinical Lumbar Spine MRI
Source: Pain Med. 2022 Oct 31;24(Suppl 1):S139–48. doi: 10.1093/pm/pnac142 (PMC10403305; doi:10.1093/pm/pnac142)
Supplement: pnac142_Supplementary_Data [file pnac142_supplementary_data.docx]

**Supplement 1: Descriptive Statistics**

Legend: Reading population reported race and smoking history descriptive statistics.

| Race |
| --- |
| 1: Native American  2: Asian  3: African American/Black  4: Pacific Islander  5: White  6: Other/Mixed  7: Prefer not to report |

**Supplement 1A:** Descriptive statistics of all 170 MR imaging exams in UCSF BACPAC dataset.

| Age | Sex (Male) | Race | Ethnicity  (Hispanic or Latino) | BMI | UBP Present | LBP Present | RBP Present |
| --- | --- | --- | --- | --- | --- | --- | --- |
| 57.8±2.25 | 45% | 1: 1%  2: 14%  3: 6%  4: 1%  5: 61%  6: 8%  7: 8%  NR: 0.0% | 7%  NR: 5% | 26.78±0.9 | 20%  NR: 15% | 73%  NR: 14% | 59%  NR: 14% |

**Supplement 1B:** Descriptive statistics of MR imaging exams on splits from Standard Split networks

|  | | | Age | Sex (Male) | Race | Ethnicity  (Hispanic or Latino) | BMI | UBP Present | LBP Present | RBP Present |
| --- | --- | --- | --- | --- | --- | --- | --- | --- | --- | --- |
| **Standard Split** | **Vert (17)** | Train  (13) | 51.17±8.79 | 15% | 2: 15%  5: 69%  6: 8%  7: 8% | 8% | 24.79±3.06 | 10% | 64% | 73% |
|  |  | Val (3) | 67.0±23.7 | 67% | 3: 33%  5: 67% | 0% | 37.13±54.27 | 33% | 67% | 100% |
|  |  | Test (1) | 58.0 | 100% | 5: 10% | 0% | 27.84 | 0% | 100% | 100% |
|  | **Disc (35)** | Train  (29) | 56.14±5.31 | 41% | 2: 10%  3: 7%  5: 76%  6: 3%  7: 3% | 3%  NR: 7% | 26.64±3.83 | 16% | 81% | 69% |
|  |  | Val  (4) | 58.75±13.71 | 25% | 5: 75%  6: 25% | 0% | 23.86±0.5 | 25% | 25% | 50% |
|  |  | Test  (2) | 69.5±120.71 | 50% | 2: 50%  5: 50% | 0% | 25.68±12.82 | NR: 100% | 100% | 100% |
|  | **Muscle (58)** | Train  (42) | 57.1±5.34 | 48% | 2: 17%  3 : 5%  5 : 67%  6 : 2%  7 : 10% | 1: 7%  2: 90%  NR: 2% | 27.28±1.88 | 23% | 90% | 72% |
|  |  | Val  (13) | 63.62±6.13 | 31% | 2: 15%  3: 8%  5: 54%  6 : 8%  7 : 15% | 1: 8%  2: 85%  NR : 8% | 25.83±3.53 | 23% | 100% | 85% |
|  |  | Test  (3) | 46.67±46.16 | 0.33% | 5: 67%  6: 33% | 0% | 27.81±48.52 | 0% | 33% | 33% |

**Supplement 1C:** Descriptive statistics of MR imaging exams on splits from Standard Split networks

|  | | | Age | Sex (Male) | Race | Ethnicity  (Hispanic or Latino) | BMI | UBP  Present | LBP Present | RBP Present |
| --- | --- | --- | --- | --- | --- | --- | --- | --- | --- | --- |
| **Shared Split** | **Vert** | Train  (10) | 56.67±9.61 | 30% | 2: 10%  3: 10%  5: 70%  7: 10% | 10%  NR: 10% | 26.66±6.13 | 12% | 78% | 78% |
|  |  | Val  (4) | 58.25±22.63 | 0% | 2: 25%  5: 50%  6: 25% | 0% | 25.61±3.56 | 0% | 67% | 67% |
|  | **Disc** | Train  (13) | 56.17±8.93 | 23% | 2: 8%  3: 8%  5: 69%  6: 8%  7: 8% | 8%  NR: 8% | 27.94±8.22 | 11% | 80% | 0.90% |
|  |  | Val  (6) | 56.17±16.51 | 50% | 2: 17%  3: 17%  5: 67% | 0% | 26.95±10.49 | 17% | 83% | 50% |
|  | **Musc** | Train  (35) | 58.4±5.92 | 43% | 2: 17%  3: 6%  5: 635  6: 3%  7: 11% | 9%  NR: 3% | 27.56±1.83 | 21% | 94% | 76% |
|  |  | Val  (14) | 58.54±8.57 | 36% | 2: 14%  3: 75  5: 50%  6: 14%  7: 14% | 7% | 26.88±4.06 | 23% | 85% | 77% |
|  | **All** | Test  (9) | 56.89  ± 10.88 | 56% | 2: 11%  5 : 89% | 0% | 25.23± 5.35 | 22% | 78% | 67% |
